# Validation of the Kihon Checklist and the frailty screening index for frailty defined by the phenotype model in older Japanese adults

**DOI:** 10.1186/s12877-022-03177-2

**Published:** 2022-06-03

**Authors:** Daiki Watanabe, Tsukasa Yoshida, Yuya Watanabe, Yosuke Yamada, Motohiko Miyachi, Misaka Kimura

**Affiliations:** 1 Faculty of Sport Sciences, Waseda University, 2-579-15 Mikajima, Tokorozawa-city, Saitama, 359-1192 Japan; 2 National Institute of Health and Nutrition, National Institutes of Biomedical Innovation, Health and Nutrition, 1-23-1 Toyama, Shinjuku-ku, Tokyo, 162-8636 Japan; 3 Institute of Interdisciplinary Research, Institute for Active Health, Kyoto University of Advanced Science, 1-1 Nanjo Otani, Sogabe-cho, Kameoka-city, Kyoto, 621-8555 Japan; 4 Senior Citizen’s Welfare Section, Kameoka City Government, 8 Nonogami, Yasumachi, Kameoka-city, Kyoto, 621-8501 Japan; 5 Physical Fitness Research Institute, Meiji Yasuda Life Foundation of Health and Welfare, 150 Tobukimachi, Hachioji-city, Tokyo, 192-0001 Japan; 6 Department of Nursing, Doshisha Women’s College of Liberal Arts, 97-1 Minamihokotate, Kodo, Kyotanabe-city, Kyoto, 610-0395 Japan; 7 Laboratory of Applied Health Sciences, Kyoto Prefectural University of Medicine, 465 Kajii-cho, Kamigyo-ku, Kyoto, 602-8566 Japan

**Keywords:** Frailty, Accuracy, Screening tool, Validation, Phenotype model

## Abstract

**Background:**

The term “frailty” might appear simple, but the methods used to assess it differ among studies. Consequently, there is inconsistency in the classification of frailty and predictive capacity depending on the frailty assessment method utilised. We aimed to examine the diagnostic accuracy of several screening tools for frailty defined by the phenotype model in older Japanese adults.

**Methods:**

This cross-sectional study included 1,306 older Japanese adults aged ≥ 65 years who underwent physical check-up by cluster random sampling as part of the Kyoto-Kameoka Study in Japan. We evaluated the diagnostic accuracy of several screening instruments for frailty using the revised Japanese version of the Cardiovascular Health Study criteria as the reference standard. These criteria are based on the Fried phenotype model and include five elements: unintentional weight loss, weakness (grip strength), exhaustion, slowness (normal gait speed), and low physical activity. The Kihon Checklist (KCL), frailty screening index (FSI), and self-reported health were evaluated using mailed surveys. We calculated the non-parametric area under the receiver operating characteristic curve (AUC ROC) for several screening tools against the reference standard.

**Results:**

The participants’ mean (standard deviation) age was 72.8 (5.5) years. The prevalence of frailty based on the Fried phenotype model was 12.2% in women and 10.3% in men. The AUC ROC was 0.861 (95% confidence interval: 0.832–0.889) for KCL, 0.860 (0.831–0.889) for FSI, and 0.668 (0.629–0.707) for self-reported health. The cut-off for identifying frail individuals was ≥ 7 points in the KCL and ≥ 2 points in the FSI.

**Conclusions:**

Our results indicated that the two instruments (KCL and FSI) had sufficient diagnostic accuracy for frailty based on the phenotype model for older Japanese adults. This may be useful for the early detection of frailty in high-risk older adults.

## Background

Frailty is a condition where multiple physiological reserves decrease as a result of the diminished ability of the stress response to cope [1, 2]. It is a public health problem among older adults worldwide [3]. Frailty is related to the risk of mortality [4, 5] or disability [4–6] in older adults. Therefore, to extend the healthy longevity of older adults, prompt detection and treatment of frailty is essential.

There are over 20 methods for assessing or screening for frailty [7]. The term “frailty” might appear simple, but the methods used to assess it differ among studies. Consequently, there is inconsistency in the classification of frailty and in the predictive capacity depending on the frailty assessment method utilised [5].

In Japan, the Kihon Checklist (KCL) [4, 8] and the frailty screening index (FSI) [6] are often used. The KCL was developed by the Japanese Ministry of Health, Labour and Welfare to screen for older adults who do not presently require care for dementia or physical disability but will require long-term care (disability) in the near future [4, 8]. Meanwhile, the FSI was developed by Yamada et al. to identify older adults who will require long-term care (disability) in the future [6]. A previous study comparing several screening tools for frailty reported that KCL was more predictive of frailty than other tools, including self-reported health, in older adults in Australia [9]. Nevertheless, to the best of our knowledge, a comparative study of the diagnostic test accuracy of several frailty screening tools for identifying frailty in older Japanese adults has not yet been conducted. This is necessary to generalise the results globally. In this study, we aimed to confirm the diagnostic accuracy of several frailty screening tools, including KCL, FSI, and self-reported health, against frailty defined by using the phenotype model in Japanese older adults. We hypothesise that the KCL and FSI more accurately screened for frailty than self-reported health and have sufficient diagnostic accuracy for frailty based on the phenotype model for older Japanese adults.

## Methods

### Study participants

Participants were selected from the cohort included in the Kyoto-Kameoka Study. The details of this study have been explained elsewhere [10–14]. Briefly, we randomly selected 10 areas from the 21 areas that make up Kameoka City in Kyoto Prefecture and mailed invitations to 4,831 residents to undergo a physical check-up. Of these residents, 1,379 participants underwent a physical check-up examination in the Kyoto-Kameoka Study between March and April 2012 (response rate: 28.5%).

This study was conducted according to the guidelines established in the 1964 Declaration of Helsinki and all procedures involving research study participants were approved by the Research Ethics Committee of Kyoto Prefectural University of Medicine (RBMR-E-363), the National Institutes of Biomedical Innovation, Health and Nutrition (NIBIOHN-76–2), and Kyoto University of Advanced Science (No. 20–1). Written informed consent was obtained from all participants prior to data acquisition.

Among the participants included in the study from baseline (*n* = 1,379), we excluded those with incomplete responses to the revised Japanese version of the Cardiovascular Health Study (revised J-CHS) criteria (*n* = 73). Ultimately, we included 1,306 participants in this study.

### Reference standards for frailty

We assessed the number of participants defined as physically frail according to the revised J-CHS criteria [15, 16]. The revised J-CHS criteria are the diagnostic criteria for physical frailty based on the Cardiovascular Health Study (CHS) that has been modified to be valid for older Japanese individuals. The revised J-CHS criteria are based on the Fried phenotype model and include five elements: unintentional weight loss, weakness (grip strength), exhaustion, slowness (normal gait speed), and low physical activity [13]. The details of the assessment of grip strength and normal gait speed have been explained elsewhere [13]. Grip strength was measured twice in each hand using a Smedley Hand Dynamometer (Grip-D TKK5101, Takei Scientific Instruments, Niigata, Japan), and the mean value of highest grip strength value for each hand was used. Gait speed was calculated as the walk a 6-m distance divided by walking time. Frailty was considered when a participant satisfied more than three of the five items of the revised J-CHS criteria. Frailty, as defined by the J-CHS criteria, has been found to predict the future risk of disability in older adults [17].

### Frailty screening tools

We used three frailty screening tools: previously validated self-administered KCL with 25 items [4, 8, 9], previously validated self-administered FSI with five items [6], and self-reported health in accordance to a previous study [9]. These questionnaires were sent via mail to the participants on 29 July 2011. The FSI mainly focuses on physical aspects (slow gait speed, cognitive domain, exhaustion, low physical activity, and weight loss) by referencing the Fried phenotype model [18]. The FSI score ranges from 0 (no frailty) to 5 (high frailty). The KCL assesses frailty from multidimensional perspectives that are similar to the deficit accumulation model [19]. Specifically, the KCL includes seven subdomains (instrumental activities of daily living disability, physical inactivity, malnutrition, oral dysfunction, socialisation domain, cognitive domain, and depression) in addition to physical aspects, and its score ranges from 0 (no frailty) to 25 (high frailty). Self-reported health was evaluated using the following questions: “How healthy do you normally feel?”, and the responses included “very healthy”, “somewhat healthy”, “not very healthy”, and “unhealthy”.

### Statistical analysis

Continuous and categorical variables are expressed as mean (standard deviation) and number (percentage), respectively. Missing values for covariates were supplemented with values from five datasets created by multivariate imputation by chained equation package using R statistical software to perform multiple imputations [20]. All missing values were assumed to be random.

To examine the validity of the screening tools, we calculated the non-parametric area under the receiver operating characteristic curve (AUC ROC) for the KCL, FSI, and self-reported health against frailty defined by the revised J-CHS criteria. The non-parametric estimate of AUC ROC was determined using the ROC model (roctab [crude] and rocreg [bootstrap]) in STATA [21]. To avoid bias due to AUC ROC calculated in the original study population, we used bootstrap to re-sample 1,000 replications to confirm the stability of the AUC ROC estimates. The AUC ROCs were compared using the ROC model (Rockcomp) in STATA [22]. We used the nearest method to find the cut-off point on the ROC curve closest to 0 for specificity and 1 for sensitivity to determine the optimal cut-off values (the point with perfect sensitivity and specificity).

The statistical significance of all statistical analyses was set at < 5% on two-tailed tests. All statistical analyses were performed using STATA MP (version 15.0; StataCorp LP, College Station, TX, USA) and R software 3.4.3 (R Core Team, Vienna, Austria).

## Results

The characteristics of the cohort analysed are shown according to sex in Table 1. The mean (standard deviation) age, body mass index, KCL score, and FSI score were 72.8 (5.5) years, 22.6 (3.3) kg/m^2^, 4.5 (3.7) points, and 1.2 (1.0) points, respectively. The prevalence of frailty based on the phenotype model was 12.2% in women and 10.3% in men.

**Table 1:** Characteristics of the Kyoto-Kameoka Study participants according to sex^a^

	Total (*n* = 1306)		Women (*n* = 656)		Men (*n* = 650)	
Age (years) ^b^	72.8	(5.5)	72.5	(5.2)	73.1	(5.8)
PD ≥ 1000 people/km^2^ (*n* [%]) ^c^	528	(40.4)	254	(38.7)	274	(42.2)
Body mass index (kg/m^2^) ^b^	22.6	(3.3)	22.3	(3.5)	22.9	(3.1)
Living alone (*n* [%]) ^c^	137	(10.5)	101	(15.4)	36	(5.5)
HSES (*n* [%]) ^c^	488	(37.4)	244	(37.2)	244	(37.5)
Education ≥ 13 y (*n* [%]) ^c^	326	(25.0)	124	(18.9)	202	(31.1)
Current smoker (*n* [%]) ^c^	104	(8.0)	14	(2.1)	90	(13.9)
Alcohol drinker (*n* [%]) ^c^	904	(69.2)	337	(51.4)	567	(87.2)
No medication (*n* [%]) ^c^	275	(21.1)	130	(19.8)	145	(22.3)
Hypertension (*n* [%]) ^c^	511	(39.1)	260	(39.6)	251	(38.6)
Stroke (*n* [%]) ^c^	36	(2.8)	12	(1.8)	24	(3.7)
Heart disease (*n* [%]) ^c^	144	(11.0)	45	(6.9)	99	(15.2)
Diabetes (*n* [%]) ^c^	118	(9.0)	46	(7.0)	72	(11.1)
Hyperlipidaemia (*n* [%]) ^c^	152	(11.6)	97	(14.8)	55	(8.5)
KCL score ^b^	4.5	(3.7)	4.4	(3.7)	4.6	(3.7)
FSI score ^b^	1.2	(1.0)	1.2	(1.0)	1.2	(1.0)
Poor self-reported health (*n* [%]) ^c,d^	164	(12.6)	77	(11.7)	87	(13.4)
Grip strength (kg) ^b^	27.7	(8.1)	33.9	(3.9)	21.5	(6.2)
Gait speed (m/s) ^b^	1.25	(0.22)	1.26	(0.21)	1.25	(0.22)
J-CHS Frailty (*n* [%]) ^c^	147	(11.3)	80	(12.2)	67	(10.3)

*FSI* Frailty screening index, *HSES* High socioeconomic status, *J-CHS* Japanese version of the Cardiovascular Health Study, *KCL* Kihon Checklist, *PD* Population density

^a^ Data for participants with missing values were imputed by multiple imputation: family structure (*n* = 67, 5.1%), socioeconomic status (*n* = 56, 4.3%), education (*n* = 116, 8.9%), smoking status (*n* = 7, 0.5%), alcohol status (*n* = 3, 0.2%), and medications (*n* = 12, 0.9%)

^b^ Continuous variables are presented as mean and standard deviation

^c^ Category variables are presented as the number of cases and percentage

^d^ Self-reported health (“very healthy” or “somewhat healthy” = good self-reported health, and “not very healthy” or “unhealthy” = poor self-reported health)

**Fig. 1:**
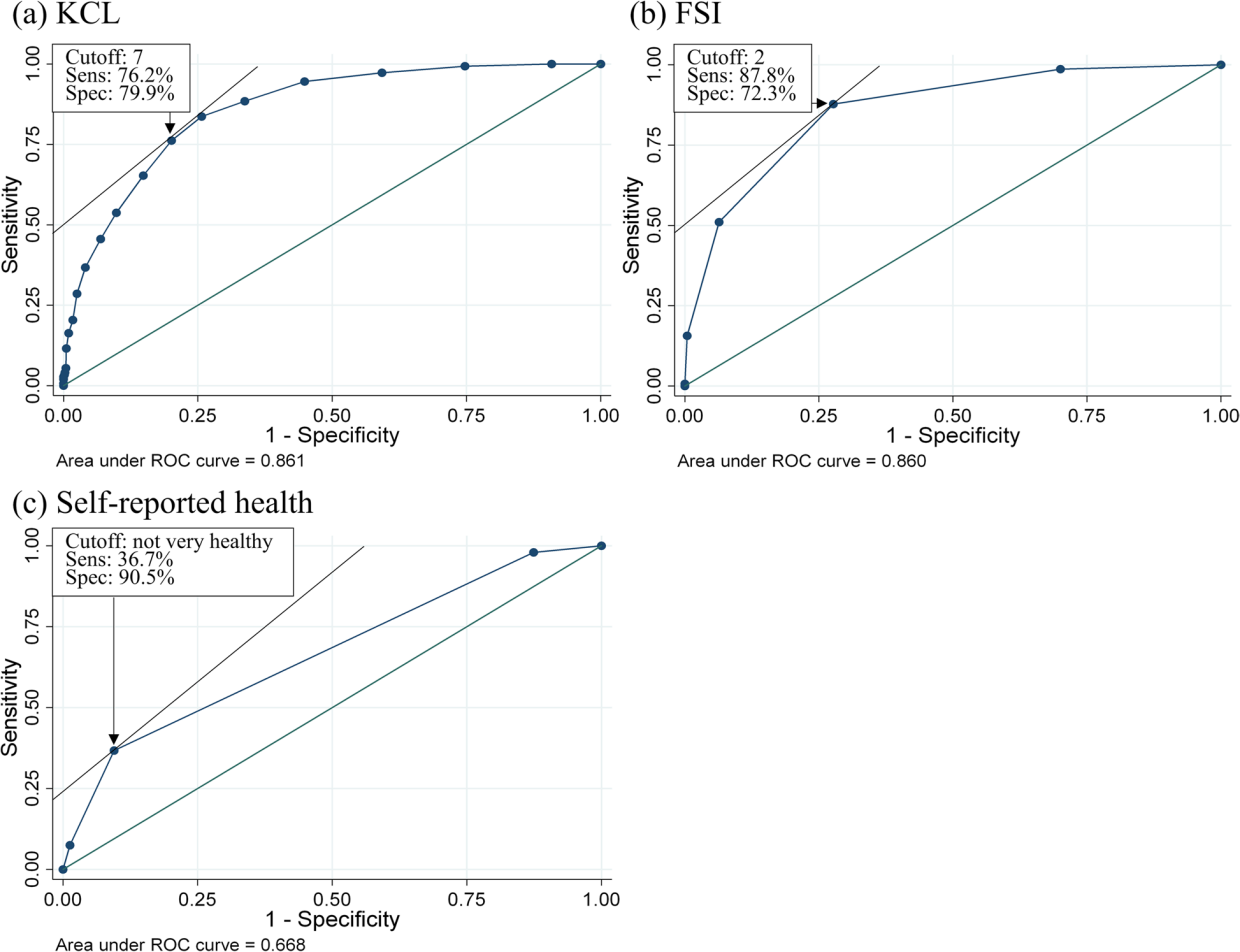
Receiver operating characteristic (ROC) curves for the Kihon Checklist (KCL), frailty screening index (FSI), and self-reported health against frailty defined by the Japanese version of the Cardiovascular Health Study criteria, which is based on the Fried phenotype model. *Sens* sensitivity, *Spec* specificity

Figure 1 and Table 2 show the validity of the KCL, FSI, and self-reported health as frailty screening tools against frailty defined by the revised J-CHS criteria, which is based on the Fried phenotype model. To evaluate the diagnostic accuracy of these tools, we calculated the AUC ROC and found it to be 0.861 (95% confidence interval [CI]: 0.832–0.889) for KCL, 0.860 (95% CI: 0.831–0.889) for FSI, and 0.668 (95% CI: 0.629–0.707) for self-reported health (Fig. 1). The predictive accuracy of self-reported health was significantly lower than that of the KCL and FSI (*p* < 0.001). The same relationship was observed when we performed the bootstrap to re-sample 1,000 replications. The cut-off for identifying frail individuals was ≥ 7 points in the KCL, ≥ 2 points in the FSI, and “not very healthy” or worse for self-reported health. Furthermore, these relationships were the same even when the results were stratified according to sex (Table 2).

**Table 2:** Validation of the Kihon Checklist, frailty screening index, and self-reported health against frailty defined by the J-CHS criteria according to the Fried phenotype model

	J-CHS criteria				Sensitivity	Specificity	PPV	NPV	LR +	LR-	AUC ROC (95% CI)			
	Frailty		Non-frailty								Crude		Bootstrap	
**KCL**														
**Total (*n* = 1306)**														
Frailty, *n*	112	(8.6)	233	(17.8)	76.2	79.9	32.5	96.4	3.8	0.3	0.861	(0.832–0.889)	0.840	(0.808–0.871)
Non-frailty, *n*	35	(2.7)	926	(70.9)										
**Women (*n* = 656)**														
Frailty, *n*	59	(9.0)	110	(16.8)	73.8	80.9	34.9	95.7	3.9	0.3	0.851	(0.809–0.892)	0.831	(0.784–0.879)
Non-frailty, *n*	21	(3.2)	466	(71.0)										
**Men (*n* = 650)**														
Frailty, *n*	53	(8.2)	123	(18.9)	79.1	78.9	30.1	97.0	3.7	0.3	0.875	(0.839–0.911)	0.856	(0.815–0.898)
Non-frailty, *n*	14	(2.1)	460	(70.8)										
**FSI**														
**Total (*n* = 1306)**														
Frailty, *n*	129	(9.9)	321	(24.5)	87.8	72.3	28.7	97.9	3.2	0.2	0.860	(0.831–0.889)	0.780	(0.728–0.833)
Non-frailty, *n*	18	(1.4)	838	(64.2)										
**Women (*n* = 656)**														
Frailty, *n*	68	(10.4)	170	(25.9)	85.0	70.5	28.6	97.1	2.9	0.2	0.837	(0.796–0.879)	0.752	(0.695–0.810)
Non-frailty, *n*	12	(1.8)	406	(61.9)										
**Men (*n* = 650)**														
Frailty, *n*	61	(9.4)	151	(23.2)	91.0	74.1	28.8	98.6	3.5	0.1	0.885	(0.846–0.925)	0.848	(0.791–0.904)
Non-frailty, *n*	6	(0.9)	432	(66.5)										
**Self-reported health**														
**Total (*n* = 1306)**														
Frailty, *n*	54	(4.2)	110	(8.4)	36.7	90.5	32.9	91.9	3.9	0.7	0.668	(0.629–0.707)	0.405	(0.336–0.473)
Non-frailty, *n*	93	(7.1)	1,049	(80.3)										
**Women (*n* = 656)**														
Frailty, *n*	27	(4.1)	50	(7.6)	33.8	91.3	35.1	90.8	3.9	0.7	0.665	(0.616–0.714)	0.407	(0.311–0.504)
Non-frailty, *n*	53	(8.1)	526	(80.2)										
**Men (*n* = 650)**														
Frailty, *n*	27	(4.2)	60	(9.2)	40.3	89.7	31.0	92.9	3.9	0.7	0.672	(0.610–0.734)	0.430	(0.335–0.526)
Non-frailty, *n*	40	(6.1)	523	(80.5)										

*AUC ROC* area under the receiver operating characteristic curve, *CI* Confidence interval, *FSI* Frailty screening index, *revised J-CHS criteria* the revised Japanese version of the Cardiovascular Health Study criteria, *KCL* Kihon Checklist, *LR +*  positive likelihood ratio, *LR − * negative likelihood ratio, *NPV* negative predictive value, *PPV* positive predictive value

The cut-off scores for diagnosing frailty is 7 points in the KCL, 2 points in FSI, and “not very healthy” or worse in self-reported health

## Discussion

In this study, we found that the KCL and FSI more accurately screened for frailty than self-reported health in older Japanese adults. To the best of our knowledge, this is the first study to compare the diagnostic accuracy of several frailty screening tools against frailty defined by the phenotype model of older Japanese adults. Our results may be useful for ensuring early detection and treatment of frailty in high-risk older adults.

Pooled analysis that included 1,755,497 participants from 240 studies reported that the prevalence of frailty in the phenotype model was 12% [23]. In addition, a previous meta-analysis has demonstrated that the prevalence of frailty in the phenotype model in Japan was 1.9%, 3.8%, 10.0%, 20.4%, and 35.1% for those aged 65–69, 70–74, 75–79, 80–84, and ≥ 85 years, respectively [24]. The results of the previous studies were similar to ours, indicating a similar prevalence of frailty. We showed that the KCL and FSI had better diagnostic accuracy for frailty than that of self-reported health; these results were similar to those of a previous study [9]. Frailty is characterised by a multidimensional factor such as psychosocial, physical, and cognitive ability playing a part in its development [1, 2]. Although the frailty in accordance to phenotype model mainly assesses frailty from the perspective of physical aspect (physical frailty) [15, 16], it may be able to insufficiently reflect the frailty condition because the self-reported health was evaluated by one item question in contrasted with screening instruments such as KCL and FSI.

It has been revealed that a KCL score of 7 or 8 points is useful for detecting frail individuals based on the Fried phenotype model [8]. Although a previous study showed a dose–response relationship between the FSI score and the incidence of disability in older Japanese adults [6], the association between the FSI score and the prevalence of frailty is unknown. Our results indicated that the KCL and FSI had similar predictive accuracies for detecting frailty in older Japanese adults. Frailty, as defined by the KCL [4] or FSI [6], is associated with risk of mortality or disability in older adults, and these screening tools may be able to detect older adults at high risk. The degree of frailty observed across most adult age groups increased in the United States between 1999 and 2018 [25]. Given that frailty can be reversed through appropriate lifestyle guidance and interventions [26, 27], our results may be useful because KCL and FSI can be used as screening tools to ensure early detection of frailty in older adults.

One of the strengths of this study was that our study population was relatively large and was selected using a cluster random sampling method. Several previous diagnostic test accuracy studies have been hindered by the limited generalisability of their results due to the absence of both sensitivity/specificity and AUC ROC [28, 29], relatively small samples [28, 30], or divergent study designs (i.e., over- or under-sampling frail individuals) [28, 30]. The current study attempted to address these issues. However, this study had certain methodological limitations. First, although we selected our study participants from among Kameoka City residents by cluster random sampling, only 28.5% underwent a physical check-up examination. Thus, these participants may have been more health-aware than the general population of older adults, opening our study to the possibility of selection bias. Second, there was an 8–9-month interval between the screening tools (mail survey) and assessment for frailty based on the Fried phenotype model (physical check-up examination). If the participant's questionnaire response includes a systematic error due to this interval, it may have served to weaken the AUC ROC of frailty screening tools against frailty defined by the phenotype model. Despite this, our study proved sufficient to confirm the predictive accuracies for these relationships. Third, we could not evaluate the diagnostic accuracy of frailty screening tools against frailty defined by the Rockwood’s frailty index based on the deficit accumulation model. A previous study used the Fried phenotype and Rockwood’s frailty index as two reference standards to verify the diagnostic accuracy of several frailty screening tools [9]. Therefore, our results must be confirmed through a well-designed study using two reference standards.

## Conclusions

The KCL and FSI had sufficient diagnostic accuracy in identifying frailty based on the phenotype model in older Japanese adults. These self-administered questionnaires can very accurately screen for frailty. With the growth of the ageing society, our results may be useful for numerous older people who may benefit from timely identification and treatment of frailty. Furthermore, it could help improve clinical practice and public health research.

## Data Availability

All data sharing and collaboration requests should be directed to the corresponding author (d2watanabe@nibiohn.go.jp), TY (t-yoshida@nibiohn.go.jp), and YY (yamaday@nibiohn.go.jp).
